# Evaluation of the Epithelial Barrier Function and Ileal Microbiome in an Established Necrotic Enteritis Challenge Model in Broiler Chickens

**DOI:** 10.3389/fvets.2018.00199

**Published:** 2018-08-21

**Authors:** Juan D. Latorre, Bishnu Adhikari, Si H. Park, Kyle D. Teague, Lucas E. Graham, Brittany D. Mahaffey, Mikayla F. A. Baxter, Xochitl Hernandez-Velasco, Young M. Kwon, Steven C. Ricke, Lisa R. Bielke, Billy M. Hargis, Guillermo Tellez

**Affiliations:** ^1^Department of Poultry Science, University of Arkansas, Fayetteville, AR, United States; ^2^Department of Food Science and Technology, Oregon State University, Corvallis, OR, United States; ^3^Department of Avian Medicine, National Autonomous University of Mexico, Mexico City, Mexico; ^4^Department of Food Science, Center of Food Safety, University of Arkansas, Fayetteville, AR, United States; ^5^Department of Animal Science, The Ohio State University, Columbus, OH, United States

**Keywords:** broiler chickens, *Clostridium perfringens*, intestinal permeability, microbiome, necrotic enteritis

## Abstract

Necrotic enteritis (NE) is a recognized multifactorial disease that cost annually to the poultry industry around $2 billion. However, diverse aspects related to its presentation are not completely understood, requiring further studies using known induction experimental models. Therefore, the purpose of this study was to measure the changes occurring in performance, intestinal integrity and ileal microbiome using a previously established NE-challenge model. Chickens were assigned to a negative control group (NC) or a positive control group (PC). In the PC, broilers were orally gavaged with *Salmonella* Typhimurium (ST) (1 × 10^7^ cfu/chick) at day 1, *Eimeria maxima* (EM) (2.5 × 10^4^ oocyst/chick) at day 18 and *Clostridium perfringens* (CP) (1 × 10^8^ cfu/chick/day) at 23–24 days of age. Weekly, body weight (BW), body weight gain (BWG), feed intake (FI) and feed conversion ratio (FCR) were evaluated. Morbidity and mortality were determined throughout the study, and NE lesion scores were recorded at day 25. Additionally, blood and liver samples were collected to measure gut permeability as determined by levels of serum fluorescein isothiocyanate-dextran (FITC-d) and bacterial translocation (BT). Ileal contents were processed for 16S rRNA gene-based microbiome analysis. Performance parameters and intestinal permeability measurements were negatively impacted in the PC resulting in elevated serum FITC-d and BT with a −6.4% difference in BWG. The NE lesion score in PC (1.97 vs. 0.00) was significantly higher in comparison to NC, although there was no difference in mortality. The microbiome analysis showed a dramatic shift of ileal microbiomes in PC groups as compared to NC (ANOSIM: *R* = 0.76, *P* = 0.001). The shift was characterized by reduced abundance of the phylum Actinobacteria (*P* < 0.01), and increased abundance of the genera *Butyrivibrio, Lactobacillus, Prevotella* and *Ruminococcus* in PC compared to NC (*P* < 0.05). Expectedly, *Clostridium* was found higher in PC (2.98 ± 0.71%) as compared to NC (1.84 ± 0.36%), yet the difference was not significant. In conclusion, results of the present study showed the different intestinal epithelial and microbiological alterations occurring in an established NE-challenge model that considers paratyphoid *Salmonella* infections in young chicks as an important predisposing factor for presentation of NE.

## Introduction

*Clostridium perfringens* (CP) is a Gram-positive anaerobe, spore-forming pathogen with a short replication rate in thioglycolate medium, and the capacity to produce more than 16 different toxins/enzymes with diverse modes of action (1–3). In mammals, it has been demonstrated that CP alpha-toxin is a key virulence factor in the pathogenesis of gas gangrene, since the injection of the alpha-toxin or injection of a beneficial *Bacillus subtilis* expressing the alpha-toxin can induce gangrene and tissue necrosis (4, 5). Alpha-toxin targets the liposomes of the cell membrane, as it contains phospholipase C and sphingomyelinase, disrupting the most important defensive organelle of the cell (6). Alpha-toxin is also responsible for hemolysis, tissue necrosis, epithelial barrier dysfunction, and severe inflammation as it activates the arachidonic acid pathway, the nuclear factor kappa beta pathway (NF-κβ), and the release of proinflammatory cytokines such as interferon gamma (IFN)-γ and tumor necrosis factor alpha (TNF)-α (7, 8). These physiological responses to alpha-toxin can also lead to edema due to increased vascular permeability (9, 10).

In chickens, CP type A and C are recognized as the primary causative agent of necrotic enteritis (NE), a multi-factorial disease that has a significant economic impact on the poultry industry with annual losses of ~2 billion dollars (11). However, even though alpha-toxin has been extensively studied in the pathogenesis of the disease (12–14), its role as the main virulent factor is no longer an accepted dogma. Few other toxins (NetB, Tpel) are now considered to be more important than alpha toxin in NE pathogenesis (3, 15). In fact, CP NetB-toxin has been also reported to induce NE without the presence of alpha-toxin (16, 17). In chickens, NE is characterized by high mortality, rapid loss in performance, depression, and a severe necrosis of the intestinal mucosa (18, 19). CP is ubiquitous and is harbored in the intestinal tract of metazoans. Hence, any condition that changes the normal microbiota (dysbacteriosis), could favor CP overgrowth and cause their toxins to rise leading to severe epithelial damage and necrosis of the intestinal absorptive surface (20–22).

Recent investigations have shown significant changes in the microbiome of chickens affected by NE when compared with healthy control chickens (23, 24). In this multi-factorial disease, coccidial infections, in particular with *Eimeria maxima* (EM) are recognized as pre-requisites in the pathogenesis of NE (11, 12). Likewise, diets with a high content of non-starch polysaccharides (NSP), immunosuppression, and withdrawal of antibiotic growth promoters or anticoccidials have been reported as factors related to the increased incidence of NE in chickens (25). The use of probiotics have been shown to reduce the incidence and severity of NE (24, 26), and these studies suggest that probiotics improve intestinal gut barrier function. However, to this date, there are no consistent results to prove this hypothesis. In an attempt to develop a reliable NE chicken model, our laboratory integrated different predisposing factors for presentation of NE that included a neonatal *Salmonella* Typhimurium (ST) challenge, followed by EM and CP challenges (27). Hence, the objectives of the present study were to evaluate the effect of NE on intestinal permeability and intestinal microbiome changes in broiler chickens in a laboratory challenge model that could be used to evaluate future alternative feed additive candidates to control the presentation of this important enteric disease.

## Materials and methods

### Animal source and diets

For the NE challenge study, a total of 80 day-of-hatch Cobb 500 male broiler chicks were obtained from a commercial hatchery (Siloam Springs, AR, USA). Chickens were neck-tagged and randomly located to one of eight floor pens (206 × 104 cm) with new pine shavings as litter in an environmentally controlled room. The temperature was maintained at 34°C for the first 5 days and was then gradually reduced until a temperature of 23°C was achieved at day 21 of age. Lighting was provided for 18 h/day. Broilers chicks were fed with mash corn-soybean based diets. Starter (0–7 days) and grower (8–25 days) diets were formulated to approximate the nutritional requirements for broiler chickens as recommended by the National Research Council (28), and adjusted to breeder's recommendations (29). Water and feed were provided *ad libitum*. No antibiotics or anticoccidials were added to the feed. In the present study, all animal handling procedures were in compliance with the University of Arkansas, Institutional Animal Care and Use Committee (IACUC, approved protocol: 15006).

### *Salmonella* typhimurium

A poultry isolate of ST selected for resistance to 25 μg/mL nalidixic acid (NA, Sigma, St. Louis, MO) and 20 μg/mL novobiocin (NO, Sigma, St. Louis, MO) was used during this study. An aliquot of ST was thawed and 100 μL of culture was inoculated into 10 mL of tryptic soy broth (TSB, Sigma, St. Louis, MO) and incubated at 37°C for 18 h. This was followed by three more passages at intervals of 8 h into fresh TSB to ensure that all bacteria were in log phase. Post-incubation, bacterial cells were washed three times in sterile saline (0.9%) by centrifugation at 1,864 × *g*, 4°C for 15 min. The approximate concentration of ST was quantified spectrophotometrically (Spectronic 20D+, Spectronic Instruments Thermo Scientific, Madison, WI) at 625 nm and diluted with sterile saline to reach a challenged concentration of ~10^7^ cfu/mL. Additionally, this concentration was also determined retrospectively, by serial dilution and plating on brilliant green agar (BGA, Sigma, St. Louis, MO) with NA (25 μg/mL) and NO (20 μg/mL) for determination of actual ST colony forming units.

### Eimeria maxima

Oocysts of the previously described EM Guelph strain (EM-GS) were donated by Dr. John. R. Barta, University of Guelph, Canada. The EM-GS strain is a single oocyst-derived isolate that has been maintained at the Ontario Veterinary College since 1973 (30). EM-GS oocysts were propagated *in vivo* in chickens experimentally inoculated with 10–30 × 10^4^ sporulated oocysts (31). The methods for detecting and recovering oocysts from infected chickens, oocyst sporulation as well as preparation of infective doses, have been previously described (32). A preliminary dose titration study was performed, offset by 1 week, to determine the EM-GS challenge dose before starting the NE study. At 13 days of age, all broilers were weighed, divided into four groups (*n* = 15/group) and challenged with three different doses (25,000, 40,000, and 50,000) of sporulated oocysts per mL by oral gavage. A group of chicks was sham challenged with saline as a negative control. Five days post-challenge, body weight (BW) and body weight gain (BWG) were recorded. Based on the criterion that the challenge dose caused sub-clinical coccidiosis, consisting of a reduction on performance parameters without the presentation of clinical signs, the lowest dose providing 25,000 oocyst per mL that caused a 24 % reduction in BWG was chosen for the present NE challenge model study. Doses corresponding to 40,000 and 50,000 oocysts per mL reduced BWG in a 27 and 28% respectively, but results were not significantly different from the lowest EM-GS challenge dose (data not shown).

### Clostridium perfringens

For CP challenge, a previously described strain used in a NE model was kindly donated and confirmed alpha-toxin positive using a multiplex PCR assay (27, 33). The primer pair used for detection of CP toxin gene *cpa* was: Forward sequence: 5′ TGCATGAGCTTCAATTAGGT 3′; Reverse sequence: 5′ TTAGTTTTGCAACCTGCTGT 3′. A frozen aliquot was amplified in TSB with sodium thioglycolate (Becton Dickinson, Sparks, MD). The broth culture was plated on phenyl ethyl alcohol agar (PEA) plates (Becton Dickinson, Sparks, MD) with 5% sheep blood (Remel, Lenexa, KS) to confirm purity. Aliquots were made with 25% sterile glycerol and stored at −80°C until further use. A single aliquot was individually amplified in TSB with sodium thioglycolate overnight for the NE challenge study and the challenge dose was confirmed by plating 10-fold dilutions on tryptic soy agar (TSA, Becton Dickinson, Sparks, MD) with sodium thioglycolate.

### Experimental design

In the NE challenge trial, 80 neonatal broiler chicks were randomly assigned to either a negative control non-challenged group (NC) or a positive control group (PC). Each experimental group had four replicates of 10 broilers. In the PC group, chickens were orally challenged with ST (1 × 10^7^ cfu/chick) at day 1, followed by EM-GS (2.5 × 10^4^ oocyst/chick) at day 18 and CP (1 × 10^8^ cfu/chick/day) at 23–24 days of age, according to a previously published experimental model (27) and the results of the *E. maxima* dose titration study described above. The NC group was sham challenged twice with saline and once with TSB with sodium thioglycolate to simulate handling and challenge conditions of the PC. Weekly, all broilers were individually weighed and BW, BWG and pen feed intake (FI) were noted at the end of each phase to calculate the feed conversion ratio (FCR) for starter (0–7 days), grower (8–25 days), and overall (0–25 days) experimental phases. Additionally, chickens were also weighed before EM-GS and CP challenge to evaluate the possible impact of each pathogen on performance. At 25 days of age, all the animals were euthanized by cervical dislocation. Chickens with similar BW where randomly selected to collect the different samples. Liver tissue was obtained for determination of aerobic and anaerobic bacterial translocation (BT) from 3 birds per pen (*n* = 12/group). In the case of the evaluation of gut permeability, blood samples were collected to measure serum fluorescein isothiocyanate-dextran levels (FITC-d) from 5 chickens per replicate (*n* = 20/group). Determination of ileal microbiome was performed based on 16S rRNA gene sequence analysis from 3 birds per pen (*n* = 12/group). CP lesion scores (*n* = 40/group) were evaluated according to Prescott et al. (34): 0 = no lesions; 1 = thin-walled and friable intestines; 2 = focal necrosis, gas production and ulceration; 3 = extensive necrosis, hemorrhagic and gas-filled intestines; and 4 = generalized necrosis typical of field cases, marked hemorrhage. Morbidity was evaluated as negative (bright and alert) or positive (reduce spontaneous activity, isolation or lethargy) according to Shojadoost et al. (35). Details about measurement techniques are described below.

### Bacterial translocation

Briefly, the right half of the liver was removed from each chicken, collected into sterile bags, weighed, homogenized, and 1:4 w/v dilutions were made with sterile 0.9% saline. Ten-fold dilutions of each sample were subsequently made in a sterile 96 well Bacti flat bottom plate, and the diluted samples were plated on TSA with and without sodium thioglycolate for evaluation of anaerobic and aerobic BT. Anaerobic samples were incubated at 37°C for 24 h using an anaerobic chamber (GasPak™, Becton Dickinson, Sparks, MD). Aerobic samples were incubated under aerobic condition using the same temperature and time parameters (37°C for 24 h). Bacterial translocation was expressed in colony forming units (Log_10_ cfu/gram of tissue).

### Determination of serum FITC-d levels

Serum levels of fluorescein isothiocyanate dextran (FITC-d), a measurement of enteric inflammation and mucosal permeability were determined after all chickens received a single oral gavage dose of FITC-d (8.32 mg/kg). Blood samples were collected from the femoral artery 1 h post FITC-d administration and allowed to clot under room temperature for 3 h. Samples were subsequently centrifuged (1,000 × *g* for 15 min) to separate serum from red blood cells. The serum samples were then diluted in phosphate buffer saline (1:5), and fluorescence was measured at 485 nm excitation and 528 nm emission (Synergy HT, multimode micro plate reader, Bio Tek Instruments, Inc., VT, USA). Levels of fluorescence in the samples were converted to respective FITC-d ng per mL of serum based on a standard curve (36).

### Preparation of the 16S rRNA gene amplicon library for MiSeq sequencing

Ileal contents (200 mg) from each bird were collected for DNA isolation utilizing QIAamp DNA Stool Mini Kit (Qiagen, Valencia, CA). The concentration of extracted DNA was diluted to 10 ng μL^−1^ for the preparation of a sequencing library targeting the V4 region of the 16S rRNA gene (37). Isolated DNA samples were amplified via a PCR using dual-index primers and normalized the amplicons with a SequalPrep™ Normalization kit (Life Technology, Carlsbad, CA) according to the manufacturers' recommendation. The library was constructed by combining 5 μL of each normalized aliquot sample for further assessment. Library concentration and product size were confirmed using a KAPA Library Quantification Kit (Kapa Biosystems, Woburn, MA) via quantitative PCR (qPCR, Eppendorf, Westbury, NY) and an Agilent 2100 Bioanalyzer system (Agilent, Santa Clara, CA), respectively. The 20 nM of pooled library aliquot and the 20 nM of PhiX control v3 were combined with 0.2 N fresh NaOH and HT1 buffer and mixed a second time with 5% of the PhiX control v3. The 600 μL of the mixture containing pooled library, PhiX control v3, NaOH and HT1 buffer was subsequently loaded onto a MiSeq v2 reagent cartridge to run sequencing.

### Analysis of microbiome sequencing data by QIIME pipeline

Raw sequencing read files were processed using quantitative insights into microbial ecology (QIIME) pipeline version 1.9.1[available at http://qiime.sourceforge.net/; (38)] at Jetstream cloud computing platform (39). Demultiplexed reads were joined together using fastq-join (40) option of QIIME. Reads that were quality filtered using multiple_split_libraries_fastq.py option of QIIME were used for chimeric sequences identification using USEARCH version 6.1.544 (41). After removing chimeric sequences, the operational taxonomic unit (OTU) picking and taxonomy assignment were performed using pick_open_reference_otus.py command of QIIME with uclust method (41). Taxonomy was assigned based on green genes taxonomy and reference database version 13_8 (42). Sequences that belong to Chloroplast and mitochondria were removed from OTU table as they are not the part of microbial communities and possible contamination of Chloroplast was previously described (43). The OTU table was normalized using cumulative sum scaling (44) before summarization and statistical comparisons of any taxa and diversity analyses. Beta diversity was calculated using weighted UniFrac metric with even sampling depth of 7,000 reads and statistical comparisons were made using analysis of similarities (ANOSIM) method.

### Statistical analysis

All data were subjected to one-way ANOVA as a completely randomized design using the GLM procedure of SAS (45). For evaluation of growth performance parameters (BW, BWG, FI, and FCR), each of the replicate pens was considered as the experimental unit (*n* = 4/group), whereas data on BT (*n* = 12/group), serum FITC-d level (*n* = 20/group) and ileal microbiome population assessment (*n* = 12/group) were based on randomly selected broilers from all replicates of each group. Treatment means were partitioned using Duncan's multiple range test at *P* < 0.05 indicating statistical significance. Mortality and morbidity were compared using the chi-square test of independence to determine significance (*P* < 0.05). Taxonomic and alpha diversity data analyzed by QIIME was imported to Microsoft Excel and JMP^®;^ Genomics 9 to determine significant differences using Wilcoxon test where level of significance was set at *P* < 0.05.

## Results

### Overall performance

The results of the evaluation of BW, BWG, FI and FCR in broiler chickens in a NE challenge model are summarized in Table 1. After 7 days of the neonatal ST challenge, there was a significant difference in BWG of 19 g between experimental groups. During the overall experimental period a significant reduction in BW (1149 vs. 854 g) and BWG (1107 vs. 811 g) were observed in the PC compared with NC group. FI was significantly reduced in the challenged group at 7 days (142 vs. 123 g) and 25 days (1754 vs. 1576 g) of age, resulting in a significant increase in the FCR from day 14–25 (1.650 vs. 2.240) and the overall experimental period in the PC compared to the unchallenged NC group (1.586 vs. 1.949).

**Table 1:** Evaluation of body weight (BW), body weight gain (BWG), feed intake (FI), and feed conversion ratio (FCR) in broiler chickens in a Necrotic enteritis challenge model^c^.

**Item**	**Negative control non-challenged**	**Positive control challenged**
**BW, g/broiler**		
day 0	42.4 ± 0.40^a^	43.1 ± 0.83^a^
day 7	148.3 ± 2.30^a^	130.1 ± 0.40^b^
day 14	390.5 ± 6.20^a^	357.5 ± 0.75^b^
day 25	1149.0 ± 11.75^a^	854.2 ± 19.60^b^
**BWG, g/broiler**		
days 0–7	106.0 ± 3.00^a^	87.0 ± 0.40^b^
days 7–14	242.3 ± 3.85^a^	227.4 ± 1.15^b^
days 14–25	758.5 ± 5.55^a^	496.8 ± 20.35^b^
days 0–25	1106.7 ± 12.15^a^	811.1 ± 18.75^b^
**FI, g/broiler**		
Days 0–7	141.6 ± 1.64^a^	123.4 ± 1.68^b^
Days 7–14	364.6 ± 2.61^a^	344.5 ± 37.50^a^
Days 14–25	1248.2 ± 2.04^a^	1114.0 ± 52.96^b^
Days 0–25	1754.3 ± 6.30^a^	1576.4 ± 86.66^b^
**FCR**		
Days 0–7	1.337 ± 0.019^a^	1.419 ± 0.026^a^
Days 7–14	1.510 ± 0.014^a^	1.517 ± 0.173^a^
Days 14–25	1.650 ± 0.010^b^	2.240 ± 0.015^a^
Days 0–25	1.586 ± 0.012^b^	1.949 ± 0.069^a^

a, b *Means in each row with different superscripts are significantly different (P < 0.05)*.

c *Data are expressed as mean ± SE; n = 40/group*.

### Body weight gain after *E. maxima* and *C. perfringens* challenge

**Figure 1:**
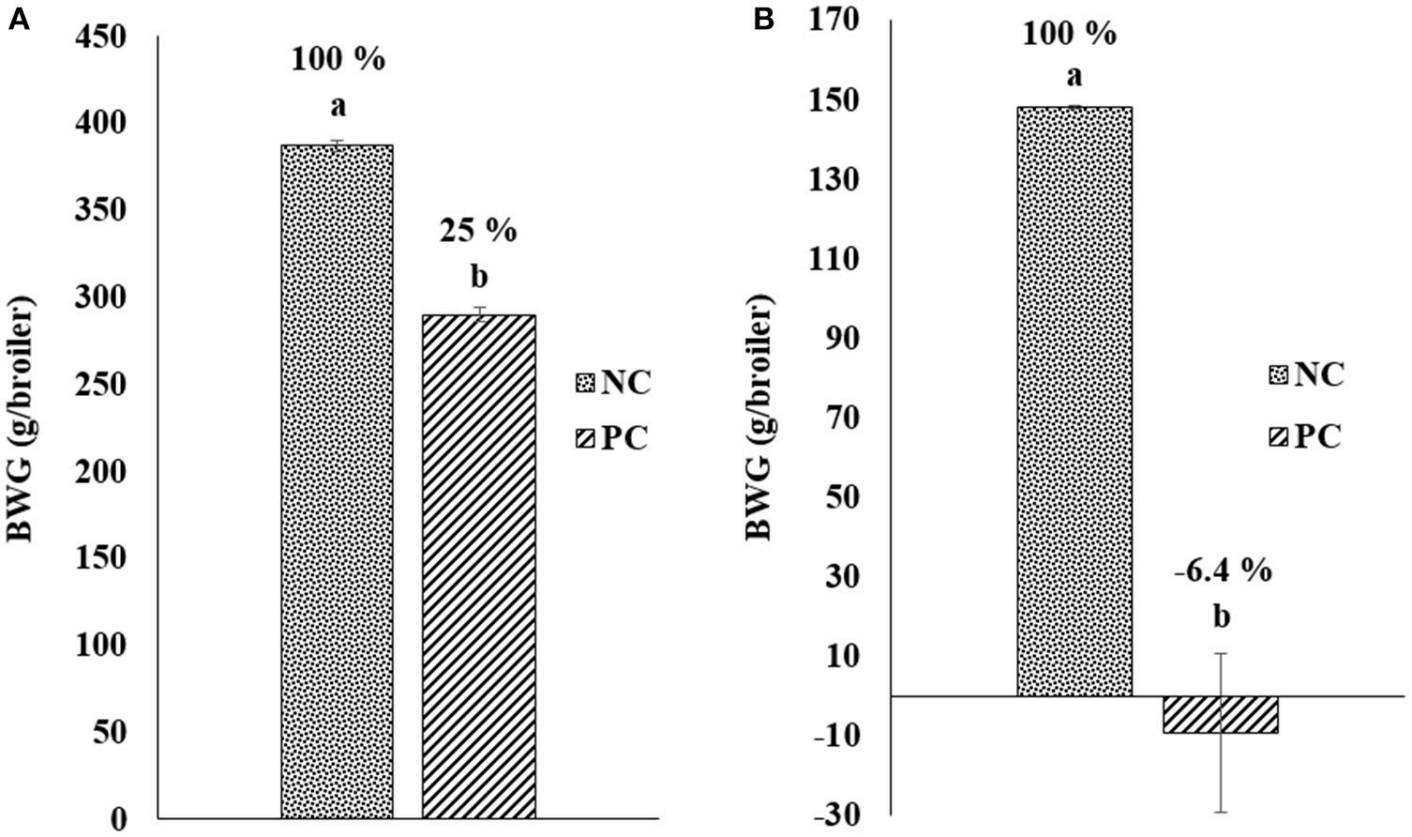
Change in body weight gain (BWG; g/broiler) after challenge with **(A)** *Eimeria maxima* and **(B)** *Clostridium perfringens* in a Necrotic enteritis model.

Figure 1 shows the results of the change in BWG after challenge with *E. maxima* and *C. perfringens* in a NE model. Chickens in PC group were weighed and challenged at day 18 of age with EM-GS (2.5 × 10^4^ oocysts/mL), and 5 days post-inoculation were weighed again to calculate the BWG difference. A reduction of 25.0% was observed in BWG in the PC with respect to non-challenged chickens in NC group (Figure 1A). Therefore, showing similar results as those of the preliminary dose titration study with the lowest 25,000 oocysts dose, resulting in a BWG difference of 24% (data not shown). Additionally, when broilers were orally administered for two consecutive days with CP, the PC showed a lost in BWG of −6.4% in comparison to the NC (Figure 1B). Therefore, the impact of CP administration in BWG was evidently higher compared to the EM-GS challenge alone, confirming the presentation of a synergistic detrimental effect on intestinal health.

### Intestinal permeability and lesion scores

Table 2 shows the results of the evaluation of gut permeability in a NE model measuring BT to the liver and serum FITC-d levels. A significant increase in both, aerobic (2.25 vs. 3.76 Log_10_ cfu/g) and anaerobic (0.61 vs. 2.28 Log_10_ cfu/g) BT to the liver tissue were observed in the PC challenge chickens when compared with the NC non-challenged chickens. Similarly, a significant increase in FITC-d leakage from the intestinal lumen to the serum was detected in challenged chickens at a magnitude 13.5-fold higher compared with unchallenged chickens (15.05 vs. 203.23 ng/mL). In the case of NE lesion scores, the NC showed no lesions (0.0), while the average lesion score for PC was 1.97, presenting focal necrosis, gas production and ulcerations in the intestinal mucosa.

**Table 2:** Evaluation of gut permeability and ileal lesion scores in a necrotic enteritis challenge model.

**Item**	**Negative control**	**Positive control**
Aerobic bacterial translocation^c^ (Log_10_ cfu/g)	2.25 ± 0.38^b^	3.76 ± 0.49^a^
Anaerobic bacterial translocation^c^ (Log_10_ cfu/g)	0.61 ± 0.49^b^	2.28 ± 0.42^a^
FITC-d^d^ (ng/mL)	15.05 ± 6.90^b^	203.23 ± 27.86^a^
Lesion Score^e^ (0–4)	0.0 ± 0.0^b^	1.97 ± 0.12^a^

a, b *Means in each row with different superscripts are significantly different (P < 0.05)*.

c *Total recovered bacteria translocated to the liver; data are expressed as mean ± SE; n = 12/group*.

d *Fluorescein isothiocyanate dextran (FITC-d); data are expressed as mean ± SE; n = 20/group*.

e *Ileal lesion score data are expressed as mean ± SE; n = 40/group*.

### Morbidity and mortality

The results of morbidity and mortality of broiler chickens in the NE model are summarized in Table 3. As expected, no mortality or clinical signs were observed in the non-challenge group. Nevertheless, at day 23 of age in the PC group, 12.5% of the chickens started to show reduced spontaneous activity (*P* < 0.05). At the second day of CP challenge, 100% of the chickens exhibited clinical signs that included either reduce activity, isolation or pronounced lethargy (*P* < 0.01). Interestingly, a significant increase in mortality was expected by 25 days of age, however, no mortality was recorded in any of the evaluated groups.

**Table 3:** Morbidity and mortality progression of broiler chickens in Necrotic enteritis model.

**Item**	**Negative control (%)**	**Positive control (%)**
Morbidity (day 22)	0	0
Morbidity (day 23)	0	12.5^*^
Morbidity (day 24)	0	100^**^
Mortality (day 25)	0	0

* *Means in each row with different superscripts are significantly different (P < 0.05); Morbidity and mortality are expressed as total percentage; n = 40/group*.

** *Means in each row with different superscripts are significantly different (P < 0.01); Morbidity and mortality are expressed as total percentage; n = 40/group*.

### Summary and comparision of significant taxa at different levels

**Figure 2:**
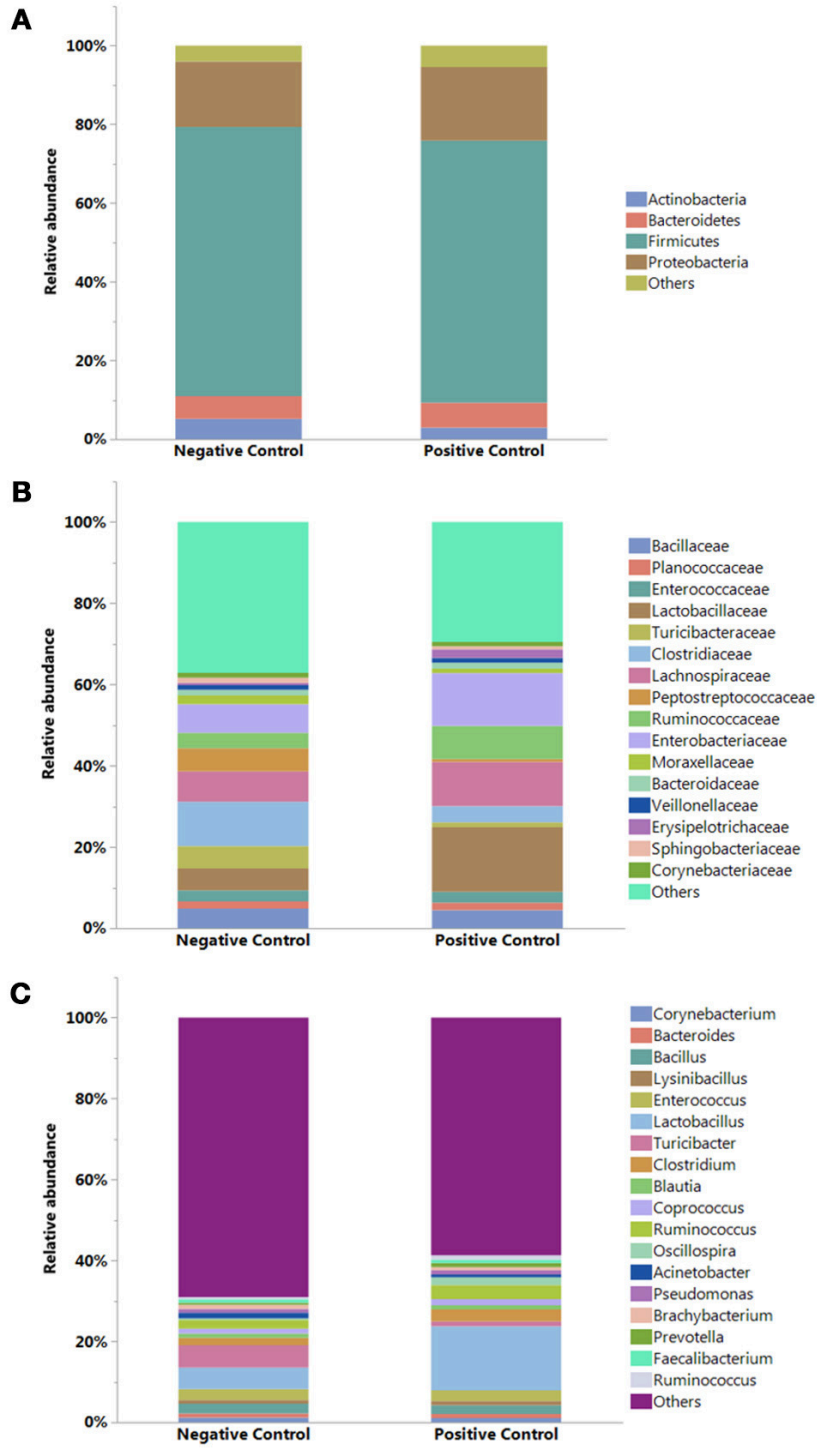
Relative abundance (%) of bacteria at **(A)** Phylum, **(B)** Family, and **(C)** Genus levels in the ileum of negative and positive control (with induced necrotic enteritis) groups. Minor bacteria genera including unassigned values were included as “others”.

The results showing relative abundance of major phyla, families, and genera are summarized in Figure 2. In addition, differentially abundant phyla, families, and genera at *P* < 0.05 are listed in Table 4. Firmicutes were found as a predominant phylum in both groups (NC; 68.46%, PC; 66.58%) followed by Proteobacteria (NC; 16.60%, PC; 18.69%), Bacteroidetes (NC; 5.72%, PC; 6.38%), and Actinobacteria (NC; 5.18%, PC; 2.91%) as shown in Figure 2A. Although, Firmicutes and Actinobacteria were found higher in NC group while Proteobacteria and Bacteroidetes were higher in PC group, significant difference was observed only with Actinobacteria (*P* < 0.01). The relative abundance of major families (≥1% in total) found on both groups is summarized in Figure 2B. In total, Lactobacillaceae (10.70%), Enterobacteriaceae (10%), Lachnospiraceae (9.20%), Clostridiaceae (7.40%), Ruminococcaceae (6%), Bacillaceae (4.7%), Turicibacteraceae (3.30%), and Peptostreptococcaceae (3.2%) were some of the major predominant families. As shown in Table 4, Brevibacteriaceae, Clostridiaceae, Flavobacteriaceae, Hyphomicrobiaceae, Microbacteriaceae, Moraxellaceae, Peptostreptococcaceae, Phyllobacteriaceae, Sphingobacteriaceae, Staphylococcaeae, and Turicibacteriaceae were significantly higher in NC group as compared to PC group (*P* < 0.05). On the contrary, Christensenellaceae, Enterobacteriaceae, Eryipelotrichaceae, Lactobacillaceae, Leuconostocaceae, Prevotellaceae, and Ruminococcaceae were significantly higher in PC group as compared to NC group (*P* < 0.05). Similarly, the relative abundance of top 18 major genera found on both groups is summarized in Figure 2C. In total, *Lactobacillus*, (10.58%), *Turicibacter* (3.33%), *Enterococcus* (2.65%), *Ruminococcus* (2.78%), *Clostridium* (2.41%), *Bacillus* (2.25%), *Coprococcus* (1.37%), and *Oscillospira* (1.19%) were predominant genera. As shown in Table 4, *Brevibacterium, Devosia, Epulopiscium, Ochrobactrum*, SMB53, and *Turicibacter* were found significantly higher in NC group as compared to PC group (*P* < 0.05). On the contrary, *Butyrivibrio, Dorea, Lactobacillus, Mogibacterium, Oscillospira, Prevotella, Proteus*, PSB-M-3, cc_115 of family Erysipelotrichaceae, and *Ruminococcus* were found significantly higher in PC group as compared to NC group (*P* < 0.05). Although not significant, *Clostridium* was found higher in PC group (2.98 ± 0.71%) as compared to NC group (1.84 ± 0.36%). Likewise, *Gallibacterium* was also found numerically higher in PC (0.79 ± 0.37%) as compared to NC (0.59 ± 0.35%).

**Table 4:** Relative abundance of phylum, family, and genera differentially present in the ileal microbiomes between negative control vs. positive control with induced necrotic enteritis^a^.

	**Negative control (%)**	**Positive control (%)**	***P*-value**
**BACTERIAL PHYLUM**			
Actinobacteria	5.18 ± 0.57	2.91 ± 0.39	0.0055
**BACTERIAL FAMILY**			
Brevibacteriaceae	0.87 ± 0.12	0.23 ± 0.09	0.0008
Christensenellaceae	0.02 ± 0.20	0.20 ± 0.06	0.0258
Clostridiaceae	10.86 ± 0.86	4.01 ± 0.80	0.0002
Enterobacteriaceae	6.98 ± 0.76	12.98 ± 1.30	0.0018
Erysipelotrichaceae	0.58 ± 0.21	2.08 ± 0.30	0.0013
Flavobacteriaceae	0.42 ± 0.10	0.08 ± 0.05	0.0118
Hyphomicrobiaceae	0.75 ± 0.13	0.01 ± 0.01	0.0002
Lactobacillaceae	5.37 ± 1.23	15.95 ± 2.34	0.0009
Leuconostocaceae	0.11 ± 0.06	0.83 ± 0.12	0.0002
Microbacteriaceae	0.34 ± 0.13	0.05 ± 0.05	0.0339
Moraxellaceae	2.15 ± 0.35	1.08 ± 0.15	0.0370
Peptostreptococcaceae	5.69 ± 0.93	0.75 ± 0.14	<0.0001
Phyllobacteriaceae	0.28 ± 0.13	0.00 ± 0.00	0.0164
Prevotellaceae	0.52 ± 0.13	1.02 ± 0.58	0.0344
Ruminococcaceae	3.83 ± 0.65	8.12 ± 1.21	0.0042
Sphingobacteriaceae	1.22 ± 0.22	0.76 ± 0.38	0.0183
Staphylococcaceae	0.97 ± 0.22	0.55 ± 0.28	0.0415
Turicibacteraceae	5.55 ± 0.71	1.11 ± 0.20	<0.0001
**BACTERIAL GENUS**			
*Brevibacterium*	0.87 ± 0.12	0.23 ± 0.09	0.0008
*Butyrivibrio*	0.08 ± 0.05	0.29 ± 0.08	0.0305
cc_115	0.08 ± 0.03	0.44 ± 0.11	0.0174
*Devosia*	0.75 ± 0.13	0.01 ± 0.01	0.0002
*Dorea*	0.50 ± 0.11	0.95 ± 0.16	0.0453
*Epulopiscium*	0.54 ± 0.15	0.03 ± 0.03	0.0039
*Lactobacillus*	5.30 ± 1.21	15.85 ± 2.28	<0.0001
*Mogibacterium*	0.00 ± 0.00	0.27 ± 0.06	0.0004
*Ochrobactrum*	0.63 ± 0.17	0.05 ± 0.04	0.0061
*Oscillospira*	0.44 ± 0.14	1.94 ± 0.63	0.0178
*Prevotella*	0.52 ± 0.13	1.02 ± 0.16	0.0344
*Proteus*	0.15 ± 0.07	0.70 ± 0.14	0.0040
PSB-M-3	0.03 ± 0.03	0.48 ± 0.08	0.0005
*Ruminococcus*	2.10 ± 0.32	3.45 ± 0.31	0.0085
SMB53	0.90 ± 0.09	0.10 ± 0.04	<0.0001
*Turicibacter*	5.50 ± 0.71	1.16 ± 0.20	0.001

a *Relative abundance data are expressed as mean ± SE; n = 12/group. Wilcoxon test was conducted to identify differentially abundant taxa where significant level was set at P < 0.05*.

### Diversity analyses

**Figure 3:**
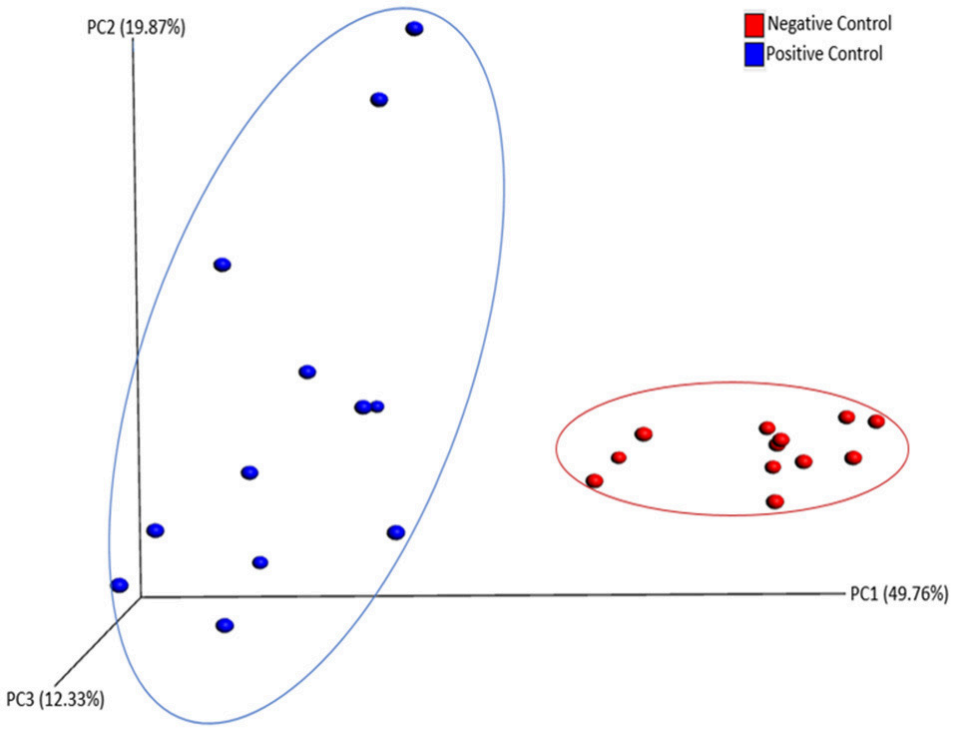
PCoA plot showing beta diversity between Negative and Positive control (with induced necrotic enteritis) as measured by weighted UniFrac metric. ANOSIM results (*R* = 0.76, *P* = 0.001) showed significant difference in community structure between groups.

There was no significant difference in alpha diversity calculated by all three metrics (chao1, PD_whole_tree, and Observed_otus available in QIIME) between NC and PC groups (data not shown). However, ANOSIM result (*R* = 0.76, *P* = 0.001) showed significant difference in beta diversity measured by weighted UniFrac metric between NC and PC groups as demonstrated in PCoA plot (Figure 3). In addition, samples in NC group were more clustered together as compared to PC group in PCoA plot, suggesting less sample wise variations existed in the microbial communities in NC group than PC group.

## Discussion

The decrease in the use of antibiotics in the poultry industry has created an opportunity for the presentation of multi-factorial diseases such as the devastating NE (46, 47). Factors that increase the presentation of this enteric disease include inappropriate management and nutritional practices or presentation of coccidiosis, leading to chronic stress and breakdown of the fragile gut microbiome. Therefore, triggering an unfavorable state of dysbacteriosis, characterized by alterations in pH, increased mucus secretion, reduced transit time and most importantly, shifting of the bacterial community (48, 49). This series of changes create ideal conditions for the rapid growth of CP, which synthesizes a collection of over 16 toxins and enzymes, most of them targeting the cell membrane of the enterocytes (1, 50). The principal CP toxins will disrupt the cell membrane by altering the cellular permeability and osmotic pressure (alpha, beta, and epsilon toxins), or by destroying the actin cytoskeleton (iota toxin) (3, 15). Interestingly, CP enterotoxin (CPE) has a different target and mode of action, as it binds the claudin family of the tight junction (TJ) proteins, causing obliteration of TJ thus increasing paracellular permeability across the enterocytes (2, 51, 52). Nevertheless, it is also crucial to mention that not all bacteria from the genus *Clostridium* are considered pathogenic. Interestingly, most of commensal *Clostridia* play decisive roles in the physiology, immunology, and even cognitive activities as some of the most important butyric acid producing bacteria of the GIT (53–57).

In the case of the current study, three different pathogens were used to successfully induce NE by disrupting the intestinal homeostasis state. The NE model included a ST challenge in neonatal broiler chickens followed by an EM oral-gavage at day 18 of age and 2 consecutive days of CP administration (27). In contrast to the high mortality and severe macroscopic lesions reported previously, the macroscopic lesions observed in PC chickens were mild, and no mortality was observed in any of the two experimental groups. However, positive control chickens were challenged with a low virulent strain of EM (Guelph strain) in contrast to the highly virulent EM (M6) used by Shivaramaiah et al. (27). Nevertheless, in the present experiment, PC challenge chickens showed a significant reduction in performance parameters; increased morbidity; and enhanced gut permeability, evidenced by liver aerobic and anaerobic BT as well as higher serum leakage of FITC-d. Alterations in gut permeability are linked with translocation of bacterial from the intestinal lumen to the portal circulation in several pathological conditions that induce leaky gut and systemic inflammation (58, 59). In the current study, PC chickens contained 13.5-fold more FITC-d in the serum when compared with NC non-challenged chickens. The relevance of this finding is that due to its large size (3–5 kDa), FITC-d does not leak into circulation under normal conditions. However, any impairment of TJ increases the permeability of FITC-d into the blood after oral administration (60–62).

The immunosuppressive effect of ST in neonatal chickens has been previously reported (63). Hence, early infection with ST in our current model has been a more reliable way to induce NE rather than using immunosuppressive viruses (18, 25). Furthermore, ST induces activation of the NF-κβ and alteration in TJ (64–66). Additionally, because the life cycle of *Eimeria* spp. involves intracellular (asexual), and extracellular (sexual) phases, a severe immune response mediated by NF-κβ activation results in the release of IFN-γ and TNF-α that contribute to the pathophysiology of coccidiosis in chickens (67, 68). Under these conditions, TNF-α increases gut permeability by downregulating TJ proteins (69–71). Furthermore, *Eimeria* infections have been also reported to induce dysbiosis. In the present study, EM challenge had a significant impact in BWG that was even more profound after the CP challenge. The increased gut permeability observed in PC group may be the result of a synergistic effect of all three pathogens involved in the NE model used in this study (ST, EM, and CP). Compromising the intestinal permeability, the largest and most important barrier against external environmental agents, the absorption of nutrients was also negatively affected, which impacted the BWG observed in the PC group as has been previously reported (72–74). Another predisposing factor to induce the presentation of NE is the utilization of cereal grains with a high content of NSP (75–77). However, in this NE study the diets provided to the animals were based on the most common feed ingredients used in the poultry industry around the world to simulate commercial conditions.

Additionally, the bacterial taxonomy results obtained in the present study are fascinating. At the phylum level, the NC group showed a higher abundance of Firmicutes and Actinobacteria as compared to PC group, however, a significant difference was observed only with Actinobacteria. Similar changes of the Actinobacteria population have been previously observed in chickens challenged with CP and coccidia in comparison to non-challenged birds (23). The most relevant observation in NC chickens regarding the Actinobacteria phylum was the significantly higher abundance of the family Brevibacteriaceae and the genus *Brevibacterium* as compared to PC chickens. Likewise, *Turicibacter*, a genus of Gram-positive bacteria that has been recognized as an important butyric acid producer was also found significantly higher in NC chickens (78, 79). The NC treatment also showed a significantly higher abundance of Peptostreptococcaceae, a family of Gram-positive bacteria in the class Clostridia that represents another important group of butyric acid-forming bacteria (80, 81).

In contrast, the PC group showed numerically higher relative abundance of Proteobacteria and Bacteroidetes compared to NC group. A similar increment in the population of Proteobacteria and Bacteroidetes has been reported before in severe cases of NE (24). The phylum Proteobacteria contain many opportunistic pathogens including bacteria from the genera *Escherichia, Salmonella, Campylobacter, and Proteus*. Therefore, an increase in Proteobacteria could be related to a probable presentation of gut dysbiosis (82). Among Proteobacteria, Enterobacteriaceae at the family level and *Proteus* at the genus level were significantly higher in PC group as compared to NC group. *Proteus* is a genus that contains opportunistic pathogens including *P. mirabilis* which has been associated with urinary tract, wound, and nosocomial infections in humans and has been considered an important zoonotic pathogen with a wider host range (83, 84). In addition, *P. mirabilis* has been isolated from chicken carcasses (85) and droppings (86), and has shown multiple drug resistance to antibiotics including tetracycline, nalidixic acid, gentamycin, and ampicillin trimethoprim-sulfamethoxazole (86).

The most notable genera among the phylum Bacteroidetes was *Prevotella*, a genus of Gram-negative bacteria which was found significantly higher in PC group compared to NC group. On the other hand, in agreement with previous findings, *Dorea* that belongs to a genus of Gram-positive bacteria of the phylum Firmicutes was significantly higher in PC group as compared to NC group (23). Similarly, *Ruminococcus*, a genus of Gram-positive bacteria associated with phylum Firmicutes and family Ruminococcaceae was significantly higher in PC group. This is in agreement with a previous NE challenged study which reported significantly higher abundance of *Ruminococcus* in the ileum of PC chickens (24). In addition, *Ruminococcus* was also found to be associated with enteritis in humans (87, 88). The association of *Ruminococcus* to enteritis in both humans and chickens may be due to its ability to utilize mucin glycans as an energy source for proliferation (89). Furthermore, a numerically higher proportion of the genus *Gallibacterium* was also observed in PC chickens as compared to NC chickens. This treatment also showed higher proportions of *Clostridium* (*P* > 0.05) and *Lactobacillus* (*P* < 0.0001). It is unclear why PC group had a higher relative abundance of *Lactobacillus*, however, it could be related to the astonishingly rapid recovery of morbid birds in the PC at day 25. Similar to our findings, *Lactobacillus* was also reported significantly higher in ceca of chickens in a different NE model that were challenged with *C. perfringens*, coccidia, and supplemented with fishmeal in the diet as compared to the non-challenged group (23). On the contrary, *Lactobacillus* was significantly higher in NC group compared to PC group in another NE challenged model study (24). On the other hand, the beta diversity analysis shows clear differences between NC and PC groups, suggesting a major shift in the gut microbiome in PC groups due to the challenges used to induce NE.

In summary, the results of the present study suggest that NE impairs the gut epithelial barrier function and induces microbiome alterations in broiler chickens in a laboratory challenge model. This additional information related to the pathogenesis and development of NE could be helpful to understand in a commercial scenario the mechanism of action of some alternative feed additives used as a replacement of antibiotic treatments to control or prevent the presentation of this important enteric disease. Studies to evaluate the dietary inclusion of a *Bacillus subtilis* direct-fed microbial (*Bacillus*-DFM) selected for production of multiple exogenous enzymes are currently in progress using the previously described NE disease model. This *Bacillus*-DFM has shown to reduce both viscosity and CP proliferation under an *in vitro* digestive system and could be one of many alternatives available to mitigate or prevent NE development without the inclusion of in-feed antibiotics (90, 91).

## Ethics statement

All animals handling procedures were in compliance with the Institutional Animal Care and Use Committee at the University of Arkansas (IACUC, approved protocol: 15006).

## Author contributions

JL, GT conceived and planned the study. JL, GT supervised all research. LB provide the *Clostridium perfringens* strain. KT, LG, BM, and MB collected data. JL, SP, GT, BA, YK, and BH analyzed and interpreted data. JL, BA, and XH-V prepared the tables and prepared the figures. JL, BA, YK, XH-V, SR, and GT wrote and/or revised the manuscript. All the authors approved the final version of the manuscript.

### Conflict of interest statement

The authors declare that the research was conducted in the absence of any commercial or financial relationships that could be construed as a potential conflict of interest.
